# Dentoalveolar changes in adults promoted by the use of auxiliary expansion arch: A cbct study

**DOI:** 10.4317/jced.56169

**Published:** 2019-10-01

**Authors:** Gustavo Siécola, José-Fernando-Castanha Henriques, Karina-Maria-Salvatore Freitas, Guilherme Janson

**Affiliations:** 1 D.D.S., M.Sc., Ph.D. Orthodontic graduate student. Department of Orthodontics, Bauru Dental School, University of São Paulo. Bauru, SP, Brazil; 2 D.D.S., M.Sc., Ph.D. Professor, Department of Orthodontics, Bauru Dental School, University of São Paulo. Bauru, SP, Brazil; 3 D.D.S., M.Sc., Ph.D. Professor, Uningá University Center, Maringá, PR, Brazil; 4 D.D.S., M.Sc., Ph.D., M.R.C.D.C. (Member of the Royal College of Dentists of Canada). Professor and Head. Department of Orthodontics. Bauru Dental School, University of São Paulo, Brazil

## Abstract

**Background:**

The objective of this study was to evaluate the dentoalveolar effects and the changes of buccal cortical bone in the posterior area after expansion obtained with TMA auxiliary expansion arch in adult patients.

**Material and Methods:**

A retrospective analysis of CT scans of 13 patients (6 male, 7 female) treated at a private clinic, taken immediately before and after the use of an auxiliary expansion archwire, was performed. Mean age at installation of TMA auxiliary expansion arch was 29.23 years (s.d.=9.13) and the mean age when the auxiliary arch was removed was 29.52 years (s.d.=9.16). Mean time of the use of the TMA auxiliary expansion arch was 0.29 years (s.d.=0.09). The patients used fixed appliances and after leveling and alignment, a TMA auxiliary expansion arch was installed, combined with the primary 0.017x0.025-inch thermoactivated Ni-Ti archwire. CBCT scans were taken at T1 and T2. Linear and angular measurements regarding the positioning of maxillary molar, premolars and canines were performed. Intragroup comparison of the variables at T1 and T2 was performed with dependent t tests.

**Results:**

There was statistically significant transverse increase and buccal inclination of all teeth. The cortical bone showed adaptability and displacement in the same direction of tooth movement, but in smaller amounts.

**Conclusions:**

The auxiliary expansion arch proved to be effective to correct dentoalveolar constriction in adult patients, by increasing the buccal dental inclination with larger displacements than the bone crest adaptation and with significant transverse gains.

** Key words:**Cone-Beam Computed Tomography, Maxillary Expansion, Adult treatment.

## Introduction

Maxillary atresia is a common malocclusion in children, adolescents and adults (1-3). The treatment of choice for this malocclusion in growing patients is rapid maxillary expansion (RME). RME is performed with devices in which forces of great magnitude are applied so that the main effects are orthopedic, thus guaranteeing significant transversal gains and reducing dental crowding (4,5).

When maxillary atresia is maintained until adulthood and therefore, without the possibility of orthopedic intervention, slow expansion of the maxilla or dentoalveolar expansion become treatment options (4,6). In this situation, devices such as Hyrax are used, respecting the protocol of slow activation, with forces of low magnitude, capable of being absorbed by the periodontal ligament (1,3,4,6-8). In addition, other devices may be used for dentoalveolar transversal expansion, such as the quad-helix, bi-helix, W-arch, all fixed to the palatal region (6).

Another method of dentoalveolar expansion described in the literature is the auxiliary expansion arch, which is a 0.6mm stainless steel wire inserted into the orthodontic accessory tube of the first maxillary molars and tied with steel ligatures between the central incisors. The purpose of this arch is to correct the posterior crossbite of the patients and expand the maxillary arch (9).

The TMA auxiliary expansion arch (titanium molybdenum alloy) is a proposal to achieve the same dentoalveolar effects of the abovementioned devices, with the advantage of presenting less discomfort to adult patients, as it is in the same position of the orthodontic leveling, as an over-arch. The choice of TMA material was due to the physical characteristics of this alloy, due to its formability and modulus of elasticity, with a more gradual release of force than steel (10-12). Another difference from this protocol is that the force is distributed throughout the leveling arch, which at this point is a 0.017x0.025-inch nickel-titanium thermoactivated archwire. In order to evaluate the torque control applied by the leveling wire present at this stage of orthodontic treatment, controlled inclination movement within the alveolar bone is obtained (2,7,10,13-15). The TMA auxiliary expansion arch (more flexibility than steel, with more gradual release of force) associated with a thermoactivated rectangular leveling arch allows control of buccal inclination movements by the rectangular wire, although with clearance in the brackets’ slot to allow for bone remodeling during orthodontic movement (16,17).

For the evaluation of dental movement in the presence of orthopedic transverse forces, there is a large literature demonstrating methodologies and results (1-4,6-8,14,18,19). Computed tomography exams illuminate complementary diagnostic methods with high-quality three-dimensional images. In orthodontics, these exams are still rarely used in daily clinical practice, being routine for some professionals, in specific cases and of greater complexity, as in canine impaction, or still used in the scope of the research (20).

Dentoalveolar expansion in adults is a widely used mechanics in cases of maxillary atresia and in cases of Class III orthodontic compensation. The technique with TMA auxiliary expansion arch has been used in clinical practice, since it is a viable option and without discomfort to the patient. However, the use of this mechanics does not present evident results in the literature. This way, the objective of this study was to evaluate the dentoalveolar effects and the changes of buccal cortical bone in the posterior area after expansion obtained with a TMA auxiliary expansion arch in adult patients.

## Material and Methods

Material

This study was approved by the Ethics in Research Committee of Bauru Dental School, University of São Paulo, Bauru, SP, Brazil. All patients read and signed informed consent forms.

The sample size calculation was based on an alpha significance level of 5% and a beta of 20% to achieve 80% test power to detect a minimum difference of 2.1° with a standard deviation of 2.47 for the maxillary first molar angulation (1). Thus, the sample size calculation showed the need for 13 patients.

The sample comprised adult patients from 18 to 44 years of age, treated in a private clinic at Bauru, SP, Brazil, by the same orthodontist. Inclusion criteria was: age above 18 years; no previous orthodontic treatment; no bracket or tube fracture or breakage during the use of the auxiliary expansion arch; presence of maxillary atresia to be treated with dentoalveolar compensation; unilateral or bilateral posterior crossbite; no gingival recession at the beginning of orthodontic treatment. Patients with skeletal posterior crossbite or severe maxillary atresia with no potential for compensatory treatment were excluded.

This way, the sample consisted of 13 patients (6 male, 7 female), with a mean age at T1 (installation of TMA auxiliary expansion arch) of 29.23 years (s.d.=9.13, minimum 18.42, maximum 51.50). The mean age when the auxiliary arch was removed (T2) was 29.52 years (s.d.=9.16, minimum 18.58, maximum 51.92). Mean usage time of the TMA auxiliary expansion arch (T2-T1) was 0.29 years (s.d.=0.09, minimum 0.16, maximum 0.42). A computed based tomography was taken at T1 and T2 for each patient.

All patients were treated by the same orthodontist, in the same private clinic, and all CBCTs were performed at the same orthodontic record center.

-Orthodontic treatment

Patients were treated with pre-adjusted, Roth prescription, self-ligating brackets (SLI, Morelli, Brazil), with the same wire sequence for leveling and alignment: 0.014, 0.018, 0.016x0.022 and 0.017x0.025-inch thermoactivated NiTi archwires. At this stage of leveling, a CBCT of the maxilla was performed, previously to installation of the expansion arch.

**Figure 1:**
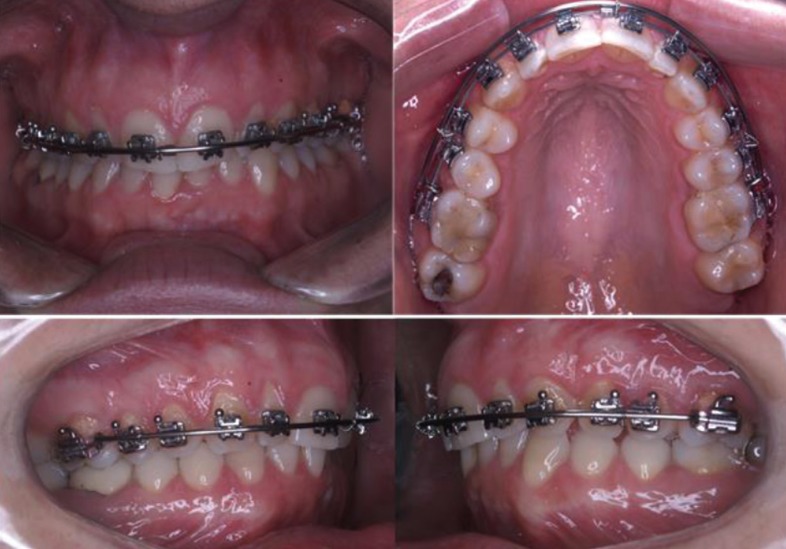
Intraoral photographs of a patient using the TMA auxiliary expansion arch.

The TMA auxiliary expansion arch was made with 0.8mm round titanium molybdenum alloy wire. Helicoids were made at 90 degrees, mesially to the first molars tubes. Thus, the main leveling wire (0.017x0.025-inch thermoactivated NiTi) would pass through these helicoids and enter the tubes of the maxillary first molars, bilaterally. In addition to these points of attachment, a 0.010-inch stainless-steel wire was also used to attach the auxiliary arch to the main leveling arch, in the region between the premolars bilaterally and between the maxillary central incisors (Fig. 1). The force released by this expansion arch was of approximately 250g.

The expansion arch was used for 2 to 3 months. The results were considered satisfactory when there was improvement of the maxillary dentoalveolar atresia, obtaining a maxillary arch shape similar to normal. The maximum limit of this dental expansion was contact of the palatal cusps of the maxillary posterior teeth with the buccal cusps of the mandibular posterior teeth. Once these results were obtained, the patient no longer used this auxiliary arch and received a leveling 0.019x0.025-inch stainless-steel archwire passively diagrammed in the new transversal dimension obtained. From this moment on, the patients received conventional orthodontic treatment and finishing.

-Methods

All patients had CBCTs performed at the same machine. The tomographic scans were taken with patients in natural head position. The tomograph was the I-CAT (Kavo), and the voxel size used was 0.25mm. Exams were saved in DICOM format, and measurements were made in OsiriX Lite software (version v.7.0.3 32-bit). The software allows multiplanar image visualization. Since only the maxilla was evaluated in CBCT, the palatal plane was used as the horizontal reference (anterior nasal spine to posterior nasal spine). Perpendicular to this plane, passing through the incisive foramen, the median vertical plane was used as a vertical reference. In coronal sectioning, for head position standardization, the palatal contour at the second molar region was used as reference, adjusting the image to be parallel to the palatal plane established in the axial section.

**Figure 2:**
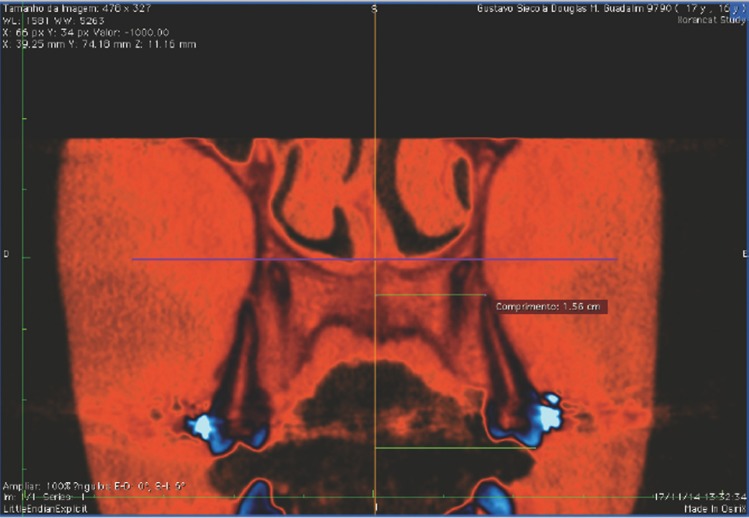
Linear measurements.

Measurements were performed through the coronal sections, marking selected points and measuring them up to the median vertical plane (MVP). Some filters were used for contrast, such as the flow and perfusion contrast. Following the orthodontic mechanics used in this study, the reference points to obtain these plans were not altered, increasing the reliability of the measurements obtained. For this research, the linear measurements were thus divided, following a previously described methodology (1):

Linear measurements (Fig. 2):

External measurements:

- Mesial and buccal cusps of the maxillary first molar to MVP (BCUSP 6)

- Buccal cusp of maxillary second premolar to MVP (BCUSP 5)

- Buccal cusp of maxillary first premolar to MVP (BCUSP 4)

- Buccal cusp of maxillary canine to MVP (BCUSP 3)

- Mesial and buccal root apex of maxillary first molar to MVP (BROOT 6)

- Buccal root apex of maxillary second premolar to MVP (BROOT 5)

- Buccal root apex of maxillary first premolar to MVP (BROOT 4)

- Buccal root apex of maxillary canine to MVP (BROOT 3)

- Buccal alveolar crest to MVP (BAC)

- Buccal alveolar crest to palatal plane vertical (BAC-PPV)

Internal measurements:

- Palatal alveolar crest to MVP (PAC)

- Palatal alveolar crest to palatal plane vertical (PAC-PPV)

**Figure 3:**
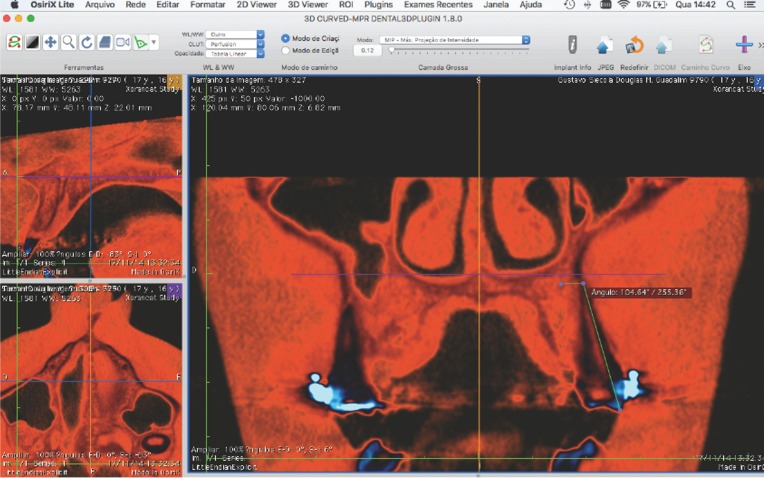
Angular measurements.

To evaluate changes in buccal inclination, angular measurements were obtained (1):

Angular measurements (Fig. 3):

- Mesial and buccal cusps of maxillary first molar to mesial root apex to MVP (ANG 6)

- Mesial cusp of maxillary second premolar to root apex to MVP (ANG 5)

- Buccal cusp of maxillary first premolar to root apex to MVP (ANG 4)

- Buccal cusp of maxillary canine to root apex to MVP (ANG 3)

Other linear measurements performed were interdental widths: intercanine, inter-first premolar, inter-second premolar and intermolar. The reference point for these measurements was the area of union of the bracket or tube to the tooth.

-Error study

Measurements were performed twice by the same operator, with interval of at least one month, in 4 subjects of the sample. Random and systematic errors were calculated by Dahlberg’s formula and dependent t tests, respectively.

-Statistical analysis

Normality of data was verified by the Kolmogorov-Smirnov test. As data presented normal distribution, parametric tests were used.

Intragroup comparison of T1 and T2 was performed by dependent t tests. Tests were performed with Statistica software (Statistica for Windows versão 7.0, Statsoft, Tulsa, Oklahoma, EUA) and were considered significant at *P*<0.05.

## Results

There were significant systematic errors in 3 variables (molar angulation, first premolar angulation and distance from palatal crest to palatal plane). The random errors varied from 0.04mm (BAC-PPV 5) to 0.25mm (I1PM) and from 0.29º (ANG 4) to 0.40º (ANG 5).

For the maxillary first molar, there was significant increase in distances BCUSP, BAC and BAC-PPV and for ANG (Table 1). For the second premolar, significant increase was observed in the variables BCUSP, BROOT, ANG and BAC ( Table 1). Regarding the first premolar, only BCUSP and ANG showed significant increases ( Table 2). For the canine, BCUSP, BROOT and ANG showed significant increases ( Table 2). All interdental widths showed significant increases ( Table 3).

**Table 1:**
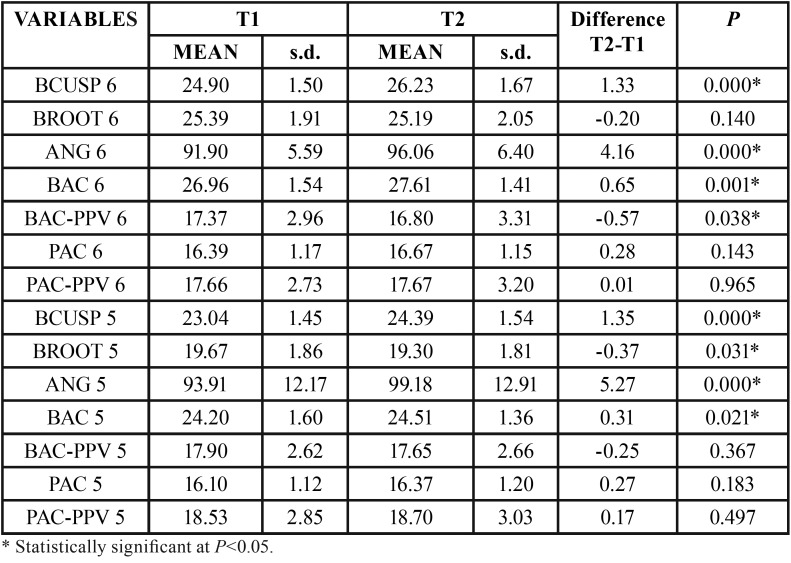
Intragroup comparison of T1 and T2 variables of maxillary first molar (6) and of maxillary second premolar (5) (dependent t test).

**Table 2:**
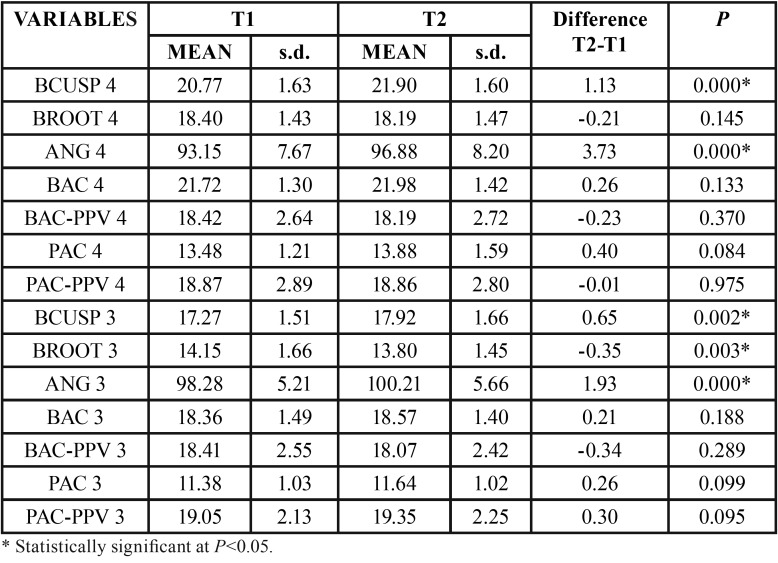
Intragroup comparison of T1 and T2 variables of maxillary first premolar (4) and of maxillary canine (3) (dependent t test).

**Table 3:**
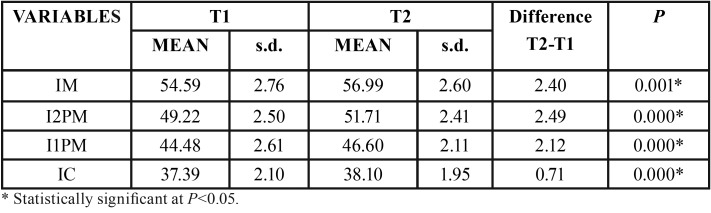
Intragroup comparison of T1 and T2 variables of interdental measurements (dependent t test).

## Discussion

-Methodology

A number of studies are currently using CT scans, especially cone-beam, in order to complete and elucidate changes during craniofacial growth (20-24), the skeletal characteristics of each facial type (14,25-27), as well as the diagnostic and therapeutic limits for orthodontics (1,3,7,18,28-33). Computed tomography is a three-dimensional exam, which gives a much broader perception of the diagnosis. It allows detection of the actual problem and identification of the individual limitations. This way, there is a need for standardization of the CT scans, especially with regard to head position. It is possible to standardize during the examination (30,32,33), or after, by adjusting the images in the interpretation software (1,7,13,26,28).

The present study used the methodology used by Baka *et al.* (1) since the main objective was to observe dentoalveolar changes. However, the quadrants were not individually evaluated because the mechanics was not asymmetrical. The use of filters allowed easier detection of skeletal limits.

-Treatment effects

The use of TMA auxiliary expansion arch in adult patients showed significant increase of the measures of cusp tips to the MVP, in all teeth evaluated, being more expressive from first premolar to molar, although for the canines it was also statistically significant ( Table 1, Table 2). It is understandable that these changes are greater in the posterior teeth, because of the design of the expansion arch, which is broader in the posterior teeth. Besides, the area of greater attachment of the auxiliary arch to the leveling wire is exactly at the molar tubes and premolar region.

In the posterior teeth, the increases in cusp tips measurements to MVP were more expressive, 1.33mm for first molars, 1.35mm for second premolars and 1.13mm for first premolars, respectively. Movement of root apices to MVP were -0.20mm, -0.37mm and 0.21mm, respectively, but statistically significant only for the second premolars ( Table 1 and  Table 2). These increases of cusp tips values are smaller than those described by Baka *et al.* (1), that were 2.15 for first molars, 2.86mm for second premolars and 3.58mm for first premolars. This can be explained because in Baka’s study (1), there was rupture of the midpalatal suture, and therefore, distance increments are also due to orthopedic effects.

Variation from T1 to T2 in canine teeth was on average 0.65mm at cusp level and -0.35 measured at the root apex, numerically small but yet statistically significant ( Table 2). This increase is associated to buccal inclination, since the mean angular increase was 1.93º. Other canine measurements did not change significantly ( Table 2). This value is slightly greater than described by Pinheiro *et al.* (34), in which the canine region did not show significant changes, even in a slow expansion with two types of fixed expansion devices and a control group.

The greater values for cusp tip measurements than for root apices are explained by the greater increase in angulation values, indicating buccal inclination. Angulation increases were of 4.16º, 5.27º and 3.73º for first molars and first and second premolars, respectively, and all were statistically significant ( Table 1, Table 2). These values for molars are close to previous results (35,36). Baka et al. (1) also showed similar buccal inclination, for premolars and first molars, but in the side that initially had crossbite; the other side without crossbite showed smaller values. These great values may be associated to the design of the present study, that used a 0.017x0.025-inch thermo-activated NiTi leveling wire, creating a significant clearance between the wire and the bracket slot (0.022x0.028-inch).

There was significant increase in the distance from the buccal alveolar crest to MVP only for first molars and second premolars, of 0.65mm and 0.31mm, on average ( Table 1). Vertically, only the buccal alveolar bone crest of first molars showed a significant decrease of 0.57mm ( Table 1). This finding is different from the literature that shows some deflection of the alveolar process in the same direction of tooth movement of around 4º (36). This magnitude of change in buccal alveolar bone crest is not the same in amount of increase of the distance from cusp tips of posterior teeth to MVP. This can be explained because this linear increase is associated to the increase in buccal inclination, and therefore with small or no bodily movement, besides the possibility of dental movement within the alveolar bone (3).

Analyzing the transversal widths, the measurements of intermolar, inter-first and inter-second premolars and intercanine distances showed mean increase of 2.40mm, 2.49mm, 2.12mm and 0.71mm, respectively ( Table 3). These increases are below the average described in the literature (3,36). However, these previous works included growing patients and evaluated rapid maxillary expansion, with rupture of the midpalatal suture (3,36). In comparison to other studies evaluating slow maxillary expansion, intermolar width increased similarly (8,19,35,37).

Therefore, the effects of the use of TMA auxiliary expansion arch are limited to dentoalveolar compensation, with dental inclinations, minor movements within the alveolar bone and slight remodeling of alveolar bone crest, primarily in the buccal region.

## Conclusions

• The effects of TMA auxiliary expansion arch are limited to dentoalveolar compensation, with dental inclinations, minor movements within the alveolar bone and slight remodeling of the bone crest, primarily in the buccal region.

• There was statistically significant transverse increase and buccal inclination of all teeth from maxillary first molar to canine. The cortical bone showed adaptability and displacement in the same direction of tooth movement, but in smaller amounts.
